# Biomarkers of Adiponectin: Plasma Protein Variation and Genomic DNA Polymorphisms

**DOI:** 10.4137/bmi.s3453

**Published:** 2009-10-13

**Authors:** Harvest F. Gu

**Affiliations:** Department of Molecular Medicine and Surgery, Karolinska Hospital, Karolinska Institutet, Stockholm, Sweden. Email: harvest.gu@ki.se

**Keywords:** adiponectin, biomarker, genetic polymorphism, protein variation

## Abstract

Adiponectin is secreted by white adipose tissue and exists as the most abundant adipokine in the human plasma. Recent research has indicated that plasma adiponectin levels are inversely correlated with body mass index (BMI) and insulin resistance. Reduction of plasma adiponectin levels is commonly observed in the patients with type 2 diabetes (T2D) and/or in those who are obese in comparison with healthy control individuals. The adiponectin (*AdipoQ*) gene has a moderate linkage disequilibrium (LD), but two small LD blocks are observed, respectively, in the promoter region and the boundary of exon 2-intron 2. Genetic association studies have demonstrated that single nucleotide polymorphisms (SNPs) +45G15G(T/G) in exon 2 and +276G/T in intron 2 of the *AdipoQ* gene confer the risk susceptibility to the development of T2D, obesity and diabetic nephropathy (DN). The SNPs in the promoter region, including −11426A/G, −11377C/G and −11391G/A, are found to be associated with T2D and DN. Recent research has indicated that the promoter polymorphisms interfere with the *AdipoQ* promoter activity. The haplotypes constructed by the promoter polymorphisms and SNP +276G/T in intron 2 are associated with circulating adiponectin levels. This review summarises genetic and pathophysiological relevancies of adiponectin and discusses about the biomarkers of adiponectin plasma protein variation and genomic DNA polymorphisms.

## Introduction

Adipose tissue or fat is a loose connective tissue composed of adipocytes. Its main role is to store triglyceride (TG) and to release free fatty acid/glycerol in response to changing energy demands. In humans, adipose tissue is located beneath the skin (subcutaneous fat), around internal organs (visceral fat), and in the bone marrow (yellow bone marrow). There exit two types of adipose tissue: white adipose tissue (WAT) and brown adipose tissue (BAT). Adipose tissue also serves as an important endocrine organ,^1^ because several hormones (adipokines) such as leptin (OMIM 164160),^2^ adipsin (OMIM 134350),^3^ tumor necrosis factor-alpha (OMIM 191160),^4^ and adiponectin (OMIM 605441),^5^ are found to be secreted by the tissue into the bloodstream. The leptin gene was identified in 1994.^6^ In one year later, adiponectin was found as the most abundant adipokine and count for 0.01% or 3–30 μg/ml of total plasma protein.^7^–^10^ In comparison with leptin research, however, until the recent 8 years, adiponectin has been attracted much attention particularly in the fields of pathophysiology and genetics of type 2 diabetes (T2D), obesity and diabetic nephropathy (DN). In this review, I will summarise genetic and pathophysiological relevancies of adiponectin and discuss about the biomarkers of its plasma protein variation and genomic DNA polymorphisms.

Adipose tissue is an endocrine organ, and adiponectin is the most abundant adipokine secreted by white adipose tissue (WAT) into the bloodstream.

## Genomic DNA Structure, mRNA and Protein of Adiponectin

**Figure 1.**
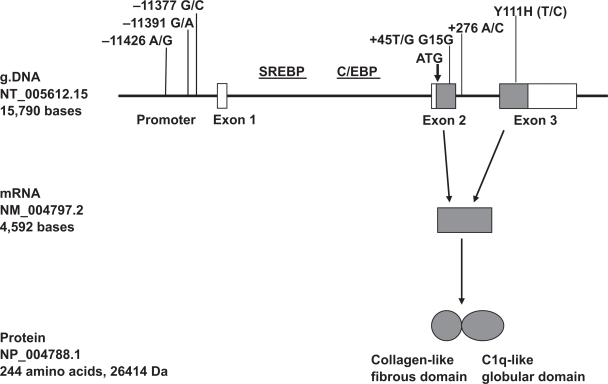
Genomic DNA, mRNA and protein of adiponectin.

The adiponectin gene (GeneID 9370) has been recently encoded by *AdipoQ* (adiponectin, C1q and collagen domain containing). The alternative names for adiponectin gene are *ACDC, APM1, ACRP30* and *GBP28*. Figure 1 demonstrates the molecular structures of genomic DNA (NT_005612.15), mRNA (NM_004797.2) and protein (NP_004788.1) of adiponectin. The *AdipoQ* gene spans 1.579 kb and contains 3 exons. The translation start point is located in exon 2. The promoter region required for the *AdipoQ* gene includes up-strand sequence, 5’-untraslated region (5’-UTR) and intron 1. The promoter region from −676 to +41 has found to be sufficient for basal transcriptional activity, in which two elements i.e. sterol regulatory binding protein (SREBP, from −431 to −423) and CCAAT/enhancer binding protein (C/EBP, from −230 to −224) reside.^11^–^19^ Adiponectin protein is constructed by 244 amino acids at a molecular weight of 26,414 Da, and consists of collagen-like fibrous and C1q-like globular domains. Sequence analysis predicted that the protein has a signal peptide but no transmembrane hydrophobic stretch, and a short N-terminal non-collagenous sequence followed by a short collagen-like motif of G-X-Y repeats. Adiponectin exists mainly as full-length in plasma. The small fragment of globular form is also found in plasma most likely due to the cleavage of adiponectin by leukocyte elastase.^20^–^22^ Northern blot analysis detected a 4.5-kb adiponectin transcript in adipose tissue but not in muscle, intestine, placenta, uterus, ovary, kidney, liver, lung, brain or heart.^23^,^24^ The *AdipoQ* mRNA in human (*Homo sapiens*) has the relatively high identical homology respectively with chimpanzee (*Pan troglodytes*, 99%), cattle (*Bos Taurus*, 87%), pig (*Sus scrofa*, 87%), dog (*Canis familiari*, 87%), mouse (*Mus musculus*, 84%) and rat (*Rattus norvegicus*, 83%). Adiponectin expression is regulated by Peroxisome proliferator-activated receptor-gamma (PPAR γ)-dependent pathways. PPAR γ ligands increase expression and plasma concentration of adiponectin.^25^–^27^

## Plasma Protein Levels and Disease Relationships for Adiponectin

There is no significant gender difference of plasma adiponectin levels in gender for newborn infants.^28^–^31^ However, plasma adiponectin levels were inversely correlated with obesity and insulin resistance in boys and girls.^32^ Adiponectin values is found to be decreased in the children whose body mass index (BMI) increased.^33^ Sex differences in adiponectin are dependent on both puberty stage and adiposity in adolescents.^34^ In adult, adiponectin expression from adipose tissue is higher in lean subjects and women, and is associated with higher degrees of insulin sensitivity.^35^

Reduction in plasma adiponectin levels are commonly observed in several metabolic disorders related with insulin resistance, including T2D and/or obesity, in both children and adults.^36^–^41^ Pima Indians in Arizona suffer from a high prevalence of T2D, which is associated with obesity. Their levels of plasma adiponectin are significantly depressed.^42^ Women with prior gestational diabetes mellitus (pGDM) are at increased risk of development of T2D, and they also had lower plasma adiponectin concentrations compared with unaffected women.^43^,^44^ Evidence has also indicated that plasma adiponectin levels independently correlate β-cell function in late pregnacy of diabetic patients. Thus, adiponectin may play a role on not only mediating insulin resistance but also β-cell dysfunction in the pathogenesis of diabetes.^45^ Furthermore, lower plasma adiponectin levels and insulin resistance coexist in a genetically prone subset of first degree African-American relatives before development of impaired glucose tolerance (IGT) and T2D with risk of diabetes.^46^ The patients with T2D not only have a decreased adiponectin levels in the basal state but also have impaired utilization of adiponectin in the coronary artery and/or the heart, which may promote the development of atherosclerosis.^47^,^48^

Plasma adiponectin levels are inversely correlated with body mass index and insulin resistance. Type 2 diabetes patients and obese subjects often have lower plasma adiponectin levels.

Interestingly, plasma adiponectin concentrations in the patients with type 1 diabetes (T1D) are found to be significantly elevated in relation to healthy controls.^49^–52^52^ In the patients with macroalbuminuria, progression to end-stage renal disease (ESRD) is found to be associated with higher serum adiponectin levels.^53^,^54^ Interrelations between adiponectin and inflammation, dyslipidemia, C-peptide levels and sex appear to be important for complex adiponectin modulation and action. The association between C-peptide and adiponectin is probably one of the reasons for their different respective levels between T1D and T2D. Development of hyperglycemia is often accompanied by dyslipidemia, hypertension heighten inflammation, endothelial dysfunction and coagulant activity, whereas adiponectin is involved in the pathophysiological mechanisms.^55^–^58^

Plasma adiponectin levels in type 1 diabetes patients and in the patients with nephropathy are increased compared to non-diabetic individuals or the patients without nephropathy. The possible mechanism concerning different adiponectin levels in type 1 and type 2 diabetes is due to adiponectin modulation and action in related to C-peptide.

Accumulating evidence shows that adiponectin plays an important role on insulin sensitivity, insulin ressistance and β-cell dysfunction. Reduction of plasma/serum adiponectin levels is significantly related to the development of diabetes, obesity and insulin resistance. Adiponectin in human serum forms a wide range of multimers from trimers and hexamers (low and/or medium molecular weight, LMW/MMW) to high molecular weight (HMW) dodecamers and 18 mers. Almost all adiponectin in plasma appears to exist as full-length adiponectin molecule.^59^–^61^ Recent research has indicated that HMW adiponectin has bioactivity. Serum HMW adiponectin is related to early postprandial glycemic increases and gastric emptying in T2D patients.^61^,^62^ However, a comparative evaluation study with the limited number of subjects (n = 60) does not support the superiority of HMW over total adiponectin in assessing metabolic variables at baseline or in response to physical training.^63^ Therefore, evaluation of serum adiponectin levels by using the ratio of HMW/(LMW + MMW) in the patients with diabetes, obesity, diabetic nephropathy and healthy individuals is of importance to better understand the role of adiponectin on the development of these complex diseases.

## Genetic Polymorphisms of the *AdipoQ* Gene and Clinically Associated with Diseases

The *AdipoQ* gene is located on chromosome 3q27. Genome wide scan studies indicate that a susceptibility locus linked to T2D, obesity and coronary heart disease may reside in this chromosomal region.^64^–^71^ Based on biological evidences and positional information from linkage studies, the *AdipoQ* gene is considered as an important candidate for T2D. Mutation screening for the *AdipoQ* gene in T2D has been done and a total of 42 single nucleotide polymorphisms (SNPs) with minor allele frequency (MAF) > 1.5% are represented in Table 1. The linkage disequilibrium (LD) of the *AdipoQ* gene is moderate, but there are two small LD blocks, one including SNPs in the promoter region and another one spanning the boundary of exon 2-intron 2.^72^ In the recent 8 years, a number of genetic association studies for the *AdipoQ* gene polymorphisms in T2D, obesity and other metabolic disorders have been reported, and the references are selected in this review. Genetic association of SNPs in the *AdipoQ* gene with T2D, obesity and DN in different ethnic populations is summarized in Table 2. There are two independent association signals, which are represented in these two LD blocks.^73^ The SNPs i.e. +45T/G and +276G/T in the *AdipoQ* gene are the common polymorphisms associated with T2D, obesity and insulin resistance.^74^–^81^ A haplotype defined by these two SNPs is found to be strongly associated with many components of insulin resistance syndrome.^73^,^82^ However, association of +45T/G and +276G/T polymorphisms with T2D is not seen in French and Swedish Caucasians. Instead, SNP −11377C/G, −11426A/G and −11377C/G in the promoter region of the *AdipoQ* gene are found to be strongly associated with T2D in French and Swedish Caucasians. 83–85 These three promoter polymorphisms are constructed as a LD block and their haplotypes are associated with type 2 diabetes and affect the promoter activity. 86 Non-synonymous SNPs in the C1q-like globular domain have relatively low MAFs (< 1.5%). H111Y is an unique non-synonymous SNP with MAF > 1.5% and found to be significantly associated with low plasma adiponectin levels, BMI and T2D.^87^ Genetic association studies have clearly demonstrated that the *AdipoQ* gene DNA polymorphisms have genetic effects on diabetes, obesity and insulin resistance, which are influenced by different genetic backgrounds and environmental factors in different ethnic populations.

Both T1D and T2D are heterogeneous disorders and the heterogeneity affects the insulin secretion and insulin action in glucose metabolism. Epidemiological studies indicate that there is the association between T1D and T2D.^88^ Adiponectin has a role on β-cell dysfunction.^89^,^90^ However, whether the *AdipoQ* gene also confers genetic susceptibility to development of T1D is unknown. A recent genetic association study of the *AdipoQ* gene in Swedish T1D patients has been performed. Results indicate that SNPs +45G15G(T/G) and +276(G/T) are strongly associated with T1D. Analyses based on haplotypic genotypes (diplotypes) constructed with these two SNPs including +45T/G and +276G/T and another two promoter SNPs i.e. −11426(A/G), −11377(G/C) indicated that common haplotypes are associated with T1D.^91^ Thus, the *AdipoQ* gene may confer the common susceptibility in the development of both T1D and T2D.

Interestingly, +276(G/T), as a putative functional SNP (pf-SNP), is found to be associated with plasma adiponectin levels in T2D,^74^ T1D^91^ and also in nonalcoholic steatohepatitis (NASH).^92^ The promoter polymorphisms, including −11426A/G, −11377C/G and −11391G/A, are also found to be associated with are associated with circulating adiponectin levels in diabetes and obesity. The haplotype with the minor allele in all three promoter polymorphisms show a complete loss of promoter activity.^83^,^85^,^86^ Thus, SNPs in the promoter and intron 2 in the *AdipoQ* gene play a functional role on the regulation of adiponectin.

Evidence indicates that the putative functional SNP (pf-SNP) +276G/T and promoter polymorphisms in the *AdipoQ* gene are associated with circulating adiponectin levels. These polymorphisms functionally regulate adiponectin promoter activity and its protein levels in blood. Knowledge of cellular mechanism how these polymorphisms regulate plasma adiponectin levels is still limited.

**Figure 2.**
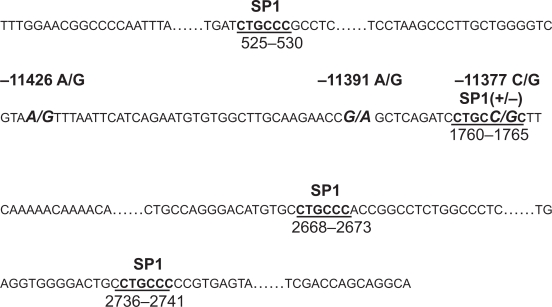
The adiponectin promoter polymorphisms and the binding sites for transcriptional factor SP1.

It has been demonstrated that SP1 belongs to the SP family of transcriptional factors, and is ubiquitously expressed in mammalian cells.^93^,^94^ Barth et al have previously reported that SP1 binding activity is enhanced during adipocyte differentiation, and has stimulatory effects on the adiponectin promoter activity.^95^ Figure 2 represents the promoter sequence and its polymorphisms in the *AdipoQ* gene. Recently, evidence has indicated that the allele G of SNP −11377C/G alters the sequence for one of four SP1 binding sites.^96^ The risk G allele results in abolishing of SP1 binding and subsequently causes reduction of adiponectin activity by ~25%.^82^ Beside SNP −11377C/G, −11391A/G is another promoter polymorphism and has moderate association with T1D diabetic nephropathy among Danish cohort but not in Finnish and French populations.^97^ Further investigation for understanding correlation between these two promoter polymorphisms and novel binding site has been taken into our consideration.

Promoter polymorphism −11377C/G resides one of transcriptional stimulatory protein (SP1) binding sites. The G allele interfere the binding function and consequently reduces the adiponectin promoter activity.

Although association of the *AdipoQ* genetic variation with plasma adiponectin levels in T2D and T1D has been well studied, further investigation is still under consideration in order to fully understand the relationship between genetic role and biological regulation of adiponectin. Indeed, regulation of plasma adiponectin levels is complex and it is under a multigenic control. Recent research has indicated that PPAR γ (P12A) and IRS-1 (G972R) genetic polymorphisms interacts the influence plasma adiponectin concentrations in young Finnish men.^98^ A linkage analysis reveals that there are two additional loci on chromosomes 14q13 and 5p15 except of the *AdipoQ* and PPAR γ genes are linked with plasma adiponectin levels.^99^ Identification of the genes in chromosomal fragments of 14q13 and 5p15 that are linked to plasma adiponectin levels may provide further information for better understanding regulation of adiponectin.

This figure is modified from Zhang et al 2009. The partial sequence from Homo sapiens adiponectin promoter (AJ011119.1, GI: 5823971) is represented. Three adiponectin promoter polymorphisms including −11426A/G, −11391G/A and −11377C/G are shown with block and italic letters. The binding sites for transcriptional factor SP1 are indicated with block letters and underline. Four binding sites for SP1 are located in g.525–530, 1760–1765, 2668–2673 and 2736–2741. SNP-11377C/G is involved in the sequence of SP1 transcriptional binding site at g.1760–1765. SP1 binding activity is enhanced during adipocyte differentiation, and has stimulatory effects on the adiponectin promoter activity (Barth et al 2002). The allele G of SNP −11377C/G alters the sequence for one of SP1 binding sites, which results in reduction of the adiponectin promoter activity by ~25% (Bouatia-Naji et al 2006).

## Summary

Taking together, biological and genetic studies have demonstrated that adiponectin plays an important role in pathogenesis of diabetes, obesity and insulin resistance. Plasma adiponectin levels are significantly decreased in T2D patients and obese subjects compared to the healthy individuals. Genetic polymorphisms in LD blocks of the *AdipoQ* gene, including the promoter region and the boundary of exon 2-intron 2, are associated with T2D, obesity, DN and insulin resistance. Therefore, plasma/serum adiponectin levels and genomic DNA polymorphisms in the *AdipoQ* gene can be used as the biomarkers for early diagnosis and clinic prediction of diabetes, obesity, diabetic complications and other metabolic disorders. Evaluation of adiponectin levels with the ratio of HMW/(LMW and MMW) and consideration of different ethnic genetic backgrounds are of importance in the translation research of adiponectin.

## Databases

OMIM: Online Mendelian Inheritance in Man http://www.ncbi.nlm.nih.gov/sites/entrez?db=OMIMdbSNP: database of Single Nucleotide Polymorphism http://www.ncbi.nlm.nih.gov/SNP/

## Figures and Tables

**Table 1.** Single nucleotide polymorphisms in the adiponectin gene.

**SNP ID**	SNP type*	**Region**	Contig position**	**Heterozygosity**
**rs16861194**	R = A/G	5′-end −11426	93054575	0.283
**rs17300539**	R = A/G	5′-end −11391	93054610	0.045
**rs266729**	S = C/G	5′-end −11377	93054624	0.368
rs182052	R = A/G	Intron 1	93055932	0.486
rs710445	R = A/G	Intron 1	93056668	0.498
rs16861205	R = A/G	Intron 1	93056784	0.251
rs16861209	M = A/C	Intron 1	93058264	0.052
rs822391	Y = C/T	Intron 1	93058953	0.160
rs822393	Y = C/T	Intron 1	93061476	0.490
rs16861210	R = A/G	Intron 1	93061648	0.131
rs822394	M = A/C	Intron 1	93061878	0.121
rs822395	M = A/C	Intron 1	93061957	0.417
rs822396	R = A/G	Intron 1	93062027	0.238
rs17366499	R = A/G	Intron 1	93062224	0.014
rs12495941	K = G/T	Intron 1	93063330	0.453
rs7649121	W = A/T	Intron 1	93063935	0.422
rs7627128	M = A/C	Intron 1	93063949	0.369
rs9877202	R = A/G	Intron 1	93064757	0.114
rs2036373	K = G/T	Intron 1	93065341	0.061
rs17366568	R = A/G	Intron 1	93065603	0.068
rs16861220	R = A/G	Intron 1	93065662	0.014
rs16861222	Y = C/T	Intron 1	93065691	0.014
rs17366653	Y = C/T	Intron 1	93065966	0.014
rs182052	R = A/G	Intron 1	93055932	0.486
rs710445	R = A/G	Intron 1	93056668	0.498
**rs2241766**	K = G/T G45G	exon 2	93066942	0.397
**rs1501299**	M = A/C	Intron 2 +276	93066273	0.426
rs2241767	R = A/G	Intron 2	93066346	0.242
rs3821799	Y = C/T	Intron 2	93066636	0.496
rs3774261	R = A/G	Intron 2	93066709	0.499
rs3774262	R = A/G	Intron 2	93066964	0.239
**rs17366743**	Y = C/T H111Y	exon 3	93067239	0.046
rs4068	Y = C/T	3′-UTR	93067708	0.014
rs1501298	Y = C/T	3′-UTR	93067792	0.393
rs6444172	M = A/C	3′-UTR	93068221	0.014
rs6444174	Y = C/T	3′-UTR	93068339	0.117
rs6773957	R = A/G	3′-UTR	93068855	0.499
rs1063537	Y = C/T	3′-UTR	93069225	0.242
rs2082940	Y = C/T	3′-UTR	93069314	0.312
rs1063538	Y = C/T	3′-UTR	93069333	0.499
rs1063539	S = C/G	3′-UTR	93070542	0.251
rs9842733	W = A/T	3′-UTR	93070632	0.074

**Notes:**

* The codes of SNP types are designed by American Association of Biochemistry.

** The contig reference ID is NT_005612.15. Data are represented from dbSNP database. Those six SNP with bold ID numbers are found to be importantly associated with metabolic disorders.

**Table 2.** Association between adiponectin genetic polymorphisms and metabolic disorders.

**SNP**	**Clinically associated**	**Ethnic group**
rs16861194	Adiponectin levels	French Caucasians, Swedish,
−11426 A/G	Type 2 diabetes	European Caucasians
rs17300539	Adiponectin levels	French Caucasians
−11391 A/G	Type 2 diabetes	UK Caucasian women
	Obesity	German, Italian
	Insulin resistance	Black South Africans
	Insulin sensitivity	Spanish, Polish
	Diabetic nephropathy	
rs266729	Adiponectin levels	French Caucasians
−11377 C/G	Type 2 diabetes	Swedish, Danish
	Obesity	German, Italian
	Insulin resistance	Black South African
	Insulin sensitivity	Chinese, Spanish
	Diabetic nephropathy	Polish
rs2241766	Adiponectin levels	Japanese, Chinese
G/T G45G	Fasting glucose levels	Korean, Italian
	Type 2 diabetes	Quebec family study
	Obesity	Uygurs, Swedish, Finnish
	Insulin resistance	African Americans
	Insulin sensitivity	Spanish, German
	Diabetic rentiopathy	European Caucasians
	Diabetic nephropathy	Polish
	Atherosclerosis	
	Cardiovascular diseases	
rs1501299	Adiponectin levels	European Caucasians
+276 A/C	Type 2 diabetes	Japanese, Italian, German
	Obesity	Chinese, Korean
	Insulin resistance	Quebec family study
	Insulin sensitivity	Swedish, Finnish
	Diabetic nephropathy	African Americans
	Polycystic ovary syndrome	Uygurs, Chinese, Spanish Polish
	Cardiovascular diseases	
rs17366743	Adiponectin levels	German, Polish
C/T H111Y	Body mass index	Finnish
	Type 2 diabetes	Framingham Americans

## Abbreviations

*AdipoQ*: adiponectin;
BMI: body mass index;
BAT: brown adipose tissue;
DN: diabetic nephropathy;
ESRD: end stage renal disease;
IGT: impaired glucose tolerance;
LD: linkage disequilibrium;
NASH: nonalcoholic steatohepatitis;
PPARγ: peroxisome proliferator-activated receptor-gamma;
pGDM: prior gestational diabetes mellitus;
PCOS: polycystic ovary syndrome;
pf-SNP: putative functional SNP;
SNP: single nucleotide polymorphism;
T1D: type 1 diabetes;
T2D: type 2 diabetes;
SP1: transcriptional stimulatory protein;
TG: triglyceride;
UTR: untraslated region,
WAT: white adipose tissue.
